# Colonic myeloid sarcoma with mutated *NPM1* manifesting with ulceration

**DOI:** 10.1002/jha2.377

**Published:** 2022-02-08

**Authors:** Mehrnoosh Tashakori, Joseph D. Khoury

**Affiliations:** ^1^ Department of Laboratory Medicine and Pathology University of Minnesota Minneapolis Minnesota USA; ^2^ Department of Hematopathology The University of Texas MD Anderson Cancer Center Houston Texas USA

**FIGURE 1:**
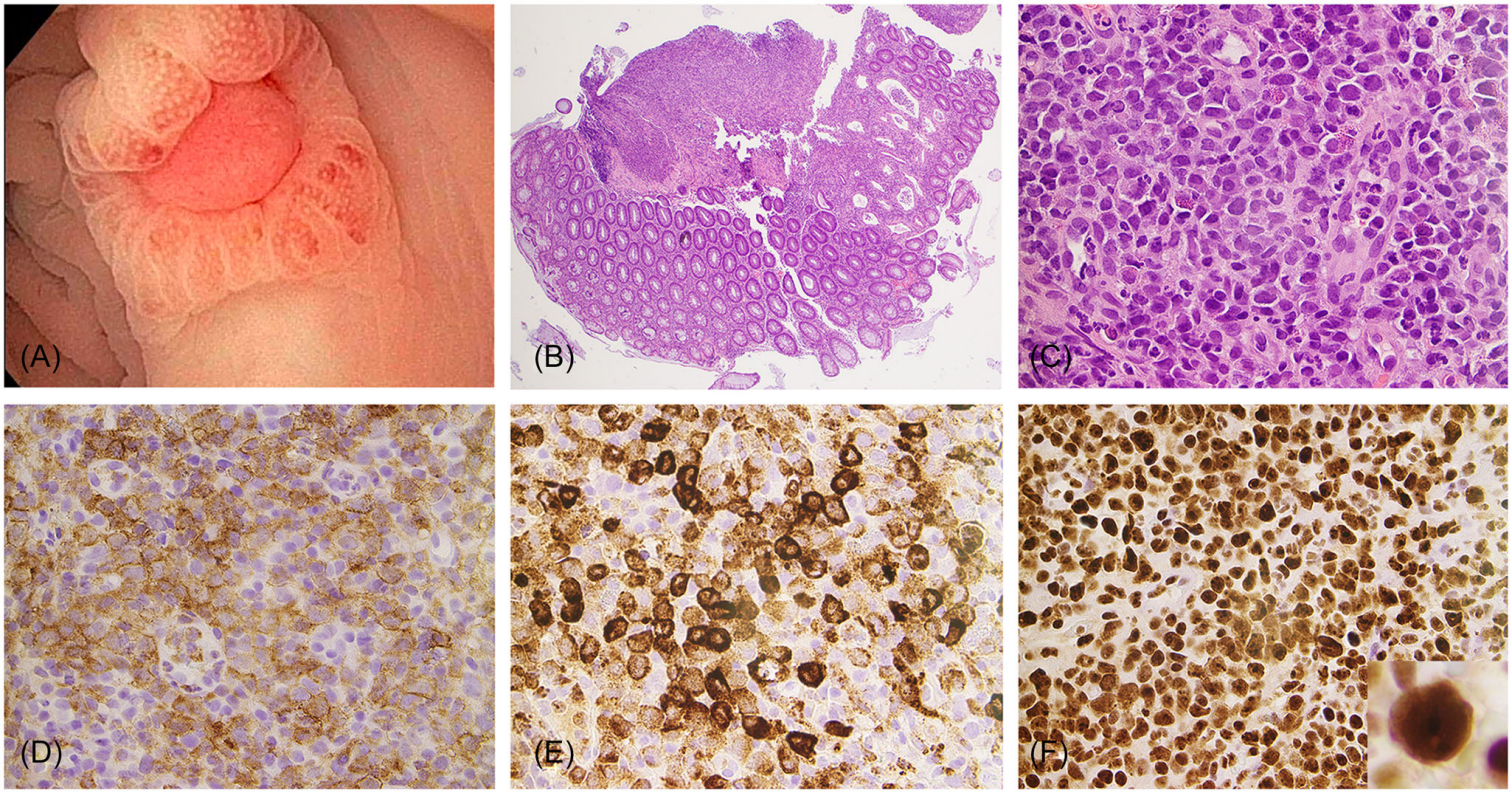
Endoscopic, histologic, and immunophenotypic findings. (A) Colonoscopy demonstrated multiple superficial ulcers. Histologic examination shows mucosal ulceration and cryptitis, notable at low‐ (B) and high‐power (C) magnification. Immunohistochemistry stains for CD33 (D) and lysozyme (E) show positive expression in immature cells, while NPM1 shows aberrant cytoplasmic signal (F and inset) within neoplastic cells

A 78‐year‐old woman presented with persistent nausea, weight loss, and fatigue. Laboratory studies revealed leukocytosis (87 ×10^9^/L) with 31% circulating blasts. Bone marrow evaluation showed acute myeloid leukemia (AML) with monocytic differentiation. Genomic profiling revealed *NPM1*p.W288fs*12, *IDH2*p.R140Q, *NRAS*p.G13D, and *PTPN11*p.P491L. During induction, the patient complained of abdominal pain. Computed tomography (CT) scan demonstrated long‐segment thickening of the rectosigmoid colon suggestive of nonspecific colitis. No mass lesions were identified. Flexible sigmoidoscopy showed multiple ulcers of variable sizes in the sigmoid, the largest being 6 mm (Figure 1A). Histopathologic evaluation showed marked mucosal ulceration and cryptitis (Figure 1B, H&E, original magnification 40×). The ulcer beds contained a polymorphous cellular infiltrate, a subset of which had immature myeloid morphology (Figure 1C, H&E, original magnification 500×) and expressed CD33 (Figure 1D, original magnification 500×) and lysozyme (Figure 1E, original magnification 500×). NPM1 immunohistochemistry revealed aberrant cytoplasmic signal (Figure 1F, original magnification 500×, inset original magnification 1000×), confirming the diagnosis of *NPM1*‐mutant myeloid sarcoma.

Colonic ulcers are a rare manifestation of myeloid sarcoma, particularly at presentation. This case highlights the importance of having a high index of suspicion for considering colonic involvement by AML in the differential diagnosis of nonspecific colitis with ulceration in patients with AML. It also shows that detection of aberrant NPM1 expression by immunohistochemistry can be a helpful adjunct in the right context.

